# Medical News

**Published:** 1866-05

**Authors:** 


### MEDICAL NEWS.

*Prof. Hamilton* is preparing a new edition of his work on Fractures and Dislocations.

*Homœpathy* has signally failed in the treatment of the rinderpest in England, where it was fairly tested.

*Obituary.*—Died, recently, in Edinburgh, Scotland, Dr. James Simpson, son of Prof. James Y. Simpson, a young physician of great promise.

*Horse-Flesh as Food.*—Markets for the sale of horse-flesh have been officially established at Paris, Vienna, Stockholm, and Copenhagen.—*N. Y. Med. Record.*

*The Cholera* has broken out with great virulence on the Rhine, and may be expected to spread over Germany, Holland, Belgium and England, during the approaching summer.

*A wealthy gentleman* of Boston has placed a sum of money in the hands of an architect, for erecting a monument in that city to commemorate the discovery of the anæsthetic properties of Ether.

*Prof. Brainard* was in Rome, with his family, on March 14, the date of his last letter to friends in Chicago. His health has improved, and he was expected to make a tour through Italy, then go to Paris.

*Dr. Mott's Will.*—The entire value of the estate of Dr. Valentine Mott is stated to be about $400,000. His anatomical museum goes by his will to the New York Medical College. —*Pacific Med. and Surg. Journal.*

*The Surgeon-General* has had constructed a beautiful model of the Hicks United States Army General Hospital at Baltimore, Md., which he designs to send to France, to be exhibited at the Paris Exposition of 1867. The model is of wood, and is made on the scale of one inch to twenty feet.

*Honors to Medical Men.*—We have heretofore announced that Prof. James Y. Simpson, of Edinburgh, has been made a Baronet. The Queen of England has further seen fit to bestow the same distinction upon William Ferguson, the eminent Surgeon of London, and Dr. Dominick Corrigan, one of the first Physicians of Dublin.—*Cin. Lancet and Observer.*

*Maximilian* has founded a Medical School in Mexico, which requires seven years to complete the course of studies; about two hundred students have been in attendance, but unless the Imperial dynasty has better fortune than some of its preceding Governments, the founder of both Empire and School will scarcely have the honor of attending its first Commencement Exercises.

*Dr. Wm. A. Hammond, Late Surgeon-General U. S. A.*—It is stated in some of the daily papers, that Dr. Hammond "has gone to Europe, in charge of a grandson of the late John Jacob Astor; and a correspondent says that he receives for his services $10,000 in gold for six months, all travelling and subsistence expenses liberally paid, and $3,000 for each month beyond the six."—*Cin. Lancet and Observer.*

*The Emperor of France* has, recently, conferred upon Dr. J. Marion Sims the Cross of the Legion of Honour, in acknowledgment of the meritorious services rendered to the great cause of Obstetrical Surgery. Dr. Sims, as is well known, first attracted public attention during the period of his residence in Montgomery, Alabama, and had, in a subsequent practice of seven years in New York City, honestly earned the distinction which was there awarded to him. He moved to Europe during the late war, and now labors professionally, both in London and Paris.

*Charivari* has a picture of the cholera, as a horrible spectre, rousing the slumbering International Commission by a fierce attack; while, on this side of the water, *Harper's Weekly* has a similar figure, foreshadowing to avaricious landlords what they may expect both in New York and Chicago.

